# Oral-Hygiene-Related Mobile Apps in the French App Stores: Assessment of Functionality and Quality

**DOI:** 10.3390/ijerph19127293

**Published:** 2022-06-14

**Authors:** Florence Carrouel, Denis Bourgeois, Céline Clément, Delphine Tardivo, Prescilla Martinon, Sébastien Guiral, Romain Lan, Stéphane Viennot, Claude Dussart, Laurie Fraticelli

**Affiliations:** 1 Health, Systemic, Process, UR 4129 Research Unit, University Claude Bernard Lyon 1, University of Lyon, 69008 Lyon, France; denis.bourgeois@univ-lyon1.fr (D.B.); celine.clement@univ-lorraine.fr (C.C.); prescilla.martinon@univ-lyon1.fr (P.M.); stephane.viennot@univ-lyon1.fr (S.V.); claude.dussart@univ-lyon1.fr (C.D.); laurie.fraticelli@univ-lyon1.fr (L.F.); 2 Prisme Team, Interpsy Laboratory, EA 4432, University of Lorraine, CEDEX, 54015 Nancy, France; 3 Laboratory Anthropology, Health Law, and Medical Ethics, UMR 7268, Aix-Marseille University 2, 13344 Marseille, France; delphine.tardivo@protonmail.com (D.T.); lanromain@live.fr (R.L.); 4 Laboratory Molecular Microbiology and Structural Biochemistry (MMSB), UMR 5086 CNRS/University of Lyon, 69367 Lyon, France; sebastien.guiral@ibcp.fr

**Keywords:** oral health, education, health promotion, mobile app, prevention

## Abstract

Mobile health apps can contribute to increased quality of individual oral hygiene behaviors. This study provides an overview and an evaluation of quality of oral-hygiene-related mobile apps currently available in Google Play Store and the French Apple App. A shortlist of nine apps was assessed by 10 oral health professionals using the Mobile App Rating Scale. Intraclass correlation was used to evaluate interrater agreement. Best quality scores were obtained by Oral-B (3.4 ± 0.97), Colgate Connect (3.20 ± 0.63), and Preventeeth (3.10 ± 1.1) and worst ones by *Mimizaur se brosse les dents* (1.80 ± 0.79) and Kolibree (2.30 ± 0.82). The subjective quality scores ranged from 2.62 ± 0.61 (Oral-B) to 1.5 ± 0.61 (MSD). Specificity of the content ranged from 3.46 ± 0.84 (Preventeeth) to 1.78 ± 0.47 (*Mimizaur se brosse les dents*). Thus, even if oral health professionals positively evaluated the quality of oral-hygiene-related mobile apps, they are less assertive concerning their impact on the user’s knowledge, attitudes, and intentions to change, as well as the likelihood of actual change in the oral hygiene behavior. Further investigations are needed to assess whether information from these apps is consistent with oral hygiene recommendations and to determine the long-term impacts of these apps.

## 1. Introduction

Oral diseases, as noncommunicable diseases, are major public health challenges [1,2]. Oral diseases worldwide account for USD 545 billion in direct and indirect costs [3]. Despite significant efforts and progress in care over the past 20 years, oral diseases remain a reality, with 3.5 billion cases of caries and 796 million people with severe periodontal disease [4]. In Europe, oral diseases represent the third highest health care expenditure compared to other noncommunicable diseases [5]. In addition, oral diseases are risk factors for noncommunicable diseases such as cardiovascular diseases, diabetes, cancer, Alzheimer’s, and adverse pregnancy outcomes [6,7,8,9,10].

Oral health is principally due to the problem of quality oral hygiene [5,11]. Oral hygiene is classically defined as an individual mechanical disorganization of dental biofilm, which has an essential role in the maintenance of good oral health [12,13]. Associated with fluoride toothpaste in children and adolescents, the rigorous control of bacterial plaque is the central element of prevention of caries [14]. Throughout life, disruption of supragingival and interdental biofilm is necessary to maintain healthy oral populations, prevent periodontal disease, and reduce the risk of systemic disease [6,15]. There is a consensus that the current unsatisfactory situation of oral health of population groups results from inadequate oral hygiene [16]. Consequently, the actors in charge of the oral hygiene education of populations cannot be limited to dentists, despite their high numbers (500,000 in Europe) [17,18], especially since the latter have also shown little interest in advocating for good oral health, preferring to treat rather than prevent oral diseases [19].

Social media brings a new dimension to health care with the possibility of potentially improving health outcomes [20]. As a new channel for health information, social media accounts could be an effective, sustainable, and feasible strategy [21]. With an ever-growing telecommunications industry, mobile technology is increasingly used for health education and assistance in behavior change around the globe for health care practice [22]. Apps for smartphones and tablets have the potential to actively educate patients by providing them with timely information through the use of push notifications [23]. There is a growing interest in mobile health apps (MHAs) for chronic diseases prevention, mostly because of their cost-effectiveness and innovations in changing behaviors [24,25]. More specifically, gamification of education, i.e., making education more playful through games, level systems, and apps for mobiles and tablets, is a sign, among other recent technological innovations, of the democratization of education and the development of new learning approaches [26]. Its importance as a tool for education is growing due to a significant and obvious change in learning preferences and practices [27].

Thus, the MHA represents a digital innovation to promote oral hygiene by educating the patient in the proper use of the equipment and the correct gestures and informing about the short- and long-term risks [28]. In a cross-sectional study, Zahid et al. (2020) compared two different oral health education approaches, conventional educational lectures and a mobile app, on oral hygiene knowledge and behavior. They concluded that the app may be a valuable tool to improve oral health knowledge, attitude, and behavior, but the quality of the app (personalization, interactivity, etc.) was critical [29]. Other studies have also concluded that the oral-health-related mobile apps improve oral hygiene [30,31,32].

By 2020, more than 350,000 health-related apps were available on the various online stores (App Store, Google Play Store, etc.). This rapid expansion of the mobile health sector makes it difficult for users to choose or to obtain advice on choosing the right app from the professionals involved [33]. However, little is known about the availability, quality, and features of mobile apps [34], including apps specifically focused on oral hygiene. The general public, patients, and health care professionals currently lack information about the uses, benefits, and limitations of mobile health for oral health communication.

The aim of this study was to provide a comparison of the quality of the oral-hygiene-related mobile apps currently available in the French Apple and Google Play stores.

## 2. Materials and Methods

### 2.1. Study Design

This study was designed as a cross-sectional study based on oral-hygiene-related mobile applications available in the French app stores. No regulatory approval was required for this study. All participants were informed of the purpose of the study and the process. This research was conducted in accordance with the STROBE guidelines (Supplementary Table S1).

### 2.2. Selection of Oral Health Professionals

Ten oral health professionals were selected (Supplementary Table S2) according to the following criteria. The inclusion criteria were (i) oral health professional or student in the final year of dental studies, and/or (ii) engaged in clinical activity in France. The exclusion criteria were (i) not having a mobile phone, (ii) not being able to download apps from the Apple or Google stores, and with (iii) hearing, visual, or motor disabilities.

### 2.3. Selection of the Oral-Hygiene-Related Mobile Apps in the French App Stores

Two university researchers made the selection of applications related to oral hygiene between 28 February and 2 March 2022. The search was conducted on the French App Store (iOS) and the French Google Play Store (Android). The words used for the search were “hygiene orale” (oral hygiene), “hygiène bucco-dentaire” (oral and dental hygiene), and “santé orale” (oral health). Due to the fact that the use of truncation or logical operators (AND, OR, and NOT) was not possible in the App Store and Google Play Store, each search term was entered separately.

Each of the two researchers eliminated duplicate apps from the same app store (iOS or Android) and established a list of apps common to both French app stores to ensure accessibility for all users. The two researchers then compared their lists to check the completeness of the lists. They then downloaded the remaining apps to their phones and checked the inclusion criteria: (1) French or English language, (2) oral hygiene topic, and (3) free apps. MHAs that could not work even partially without the addition of extra hardware (electric toothbrush., etc.) were excluded.

### 2.4. Evaluation of the Oral-Hygiene-Related Mobile Apps in the French App Stores

#### 2.4.1. Use of the Standardized Rating Scale for Mobile Applications

The French version of the Mobile App Rating Scale (MARS-F) [35] adapted from the original English version MARS was used [36]. The first part of the MARS scale, called “App classification”, was reviewed by the two academic researchers. The MARS scale used to evaluate health-related mobile apps consists of a main part (23 items divided into 5 sections called A, B, C, D, and E) and an additional part (6 items in section F) [36,37,38,39].

The engagement section (section A, 5 items) assesses whether the app is fun, interesting, customizable, and interactive (e.g., sends alerts, messages, reminders, feedback, allows sharing). The functionality section (section B, 4 items) focuses on how the app works, how easy it is to learn, how easy it is to use, how to send alerts, messages, reminders, feedback, and the ability to share information. The aesthetics section (section C, 3 items) analyzes the app’s graphic design, overall visual appeal, color palette, and stylistic consistency. The information quality section (section D, 7 items) determines whether the app contains high-quality information (e.g., text, comments, measurements, and references) from a credible source. The subjective section (section E, 4 items) assesses the user’s interest in the application. Section F, the last section of MARS, analyzes the point of view of oral health professionals regarding the effect of the selected apps on knowledge, possible changes in user attitudes and intentions to change, and the probability of changing the identified targeted behaviors. For this section, it is necessary to determine the health behavior targeted by the apps evaluated, so in our study, it was “daily oral hygiene habits”.

Each item was evaluated using a 5-point Likert scale anchored by 1 = strongly disagree and 5 = strongly agree. Each section is evaluated by the mean score of items. The overall quality MARS score is the mean of the sections scores A, B, C, and D as documented in Stoyanov et al. [36]. Sections E, related to the subjective quality, and F, related to specificities of the app, are not included in the overall quality MARS score. A score of 1 (minimum score) indicates poor quality while a score of 5 (maximum score) indicates high quality.

#### 2.4.2. Evaluation of the Apps by Raters

Ten oral health professionals carried out the app evaluation (Supplementary Table S2). Before beginning to evaluate apps related to oral hygiene, raters had to be trained to use the MARS-F scale. For this purpose, all raters had at their disposal a French training video (available on request from the corresponding author) developed for the MARS-F grid [35] and adapted from the English training video by Stoyanov et al. [36]. This video presented each item and the answers with examples. At the end of the video, a training exercise was proposed with an app not included in the sample of the study. All raters had to evaluate the same app (8fit) that focused on physical activity and not oral hygiene. The raters downloaded, tested the app for at least 10 min, and then completed the MARS-F questionnaire. Then, the raters compared the results with the ones in the video. If the individual assessment score varied by at least 2 points, raters discussed until consensus to ensure a similar understanding of the item.

The oral-hygiene-related apps were evaluated by the 10 raters during the month of March 2022. The raters downloaded all the applications. Then, they used each application for 10 min before evaluating it using the standardized online MARS-F questionnaire.

Three of the ten raters had already used the Oral-B app for personal use before testing it for the study, but all the three raters were no longer using it at the time of the study. All these three raters would recommend it for their patients or have already recommended it.

### 2.5. Statistical Analysis

The intraclass correlations (ICC) (two-way random, average measures, absolute concordance) were calculated to assess interrater reliability [40,41]. The 95% confidence intervals were calculated for each item, and for each section and for the overall quality MARS score (ABCD sections). Based on the 95% confidence interval of the ICC estimate, values less than 0.5, between 0.5 and 0.75, between 0.75 and 0.9, and greater than 0.90 are indicative of poor, moderate, good, and excellent reliability, respectively [41]. Mean values and standard deviations were calculated per item and per MHA section (noted in parentheses, mean ± standard deviation). Item 19 was excluded from all analyses due to missing values (N/A: not applicable), and the mean for section D was adjusted accordingly.

In order to compare the differences between the quality of the applications, by item and by section, box plots were produced.

A heatmap was constructed to represent an overview of the average scores per item (row) and per app (column). The color gradients indicated whether the score was low, near 1 (yellow), or high, near 5 (green).

The correlation between the average quality and the subjective item 23 (“What is your overall star rating of the application?”) was assessed using the Pearson coefficient (r). The number of star rating from iOS and Android store was reported to provide a comprehensive overview of the popularity of each mobile app, and also the number of raters.

Statistical analyses were performed using R using the “dplyr”, ”psych”, and “ggplot2” packages from the R Project for Statistical Computing (version 4.1.1. 2021-08-10).

## 3. Results

### 3.1. Selection of the Oral-Hygiene-Related Mobile Apps

**Figure 1:**
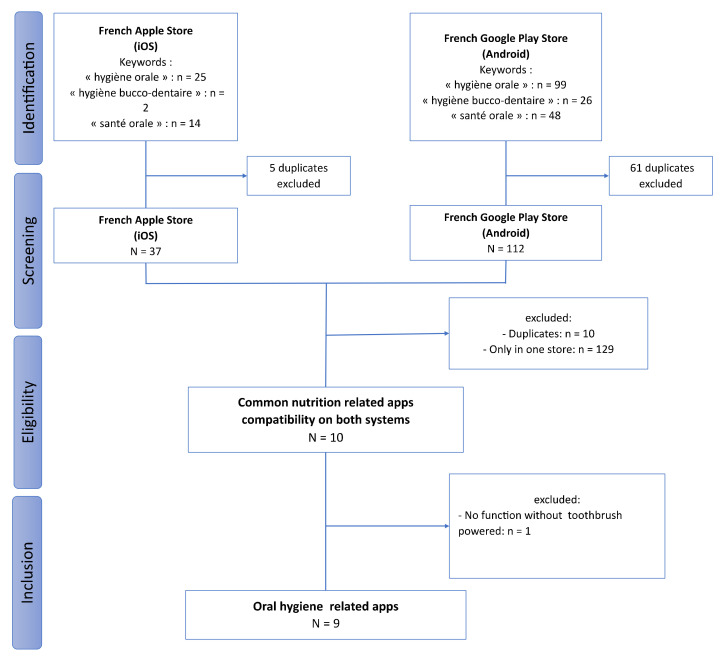
Flowchart of the oral-hygiene-related mobile apps selection.

A total of 41 apps in the App Store and 173 apps in the Google Play Store were identified (Figure 1). The duplicates were eliminated in each list. The two lists were cross-checked, analyzing the name of the app and the developer. Ten apps were available and common on both systems. After downloading, one app was excluded due to the fact that it was unusable without a powered toothbrush device. Finally, nine apps were included.

### 3.2. Characteristics of the Oral-Hygiene-Related Mobile Apps

Technical and descriptive information about the oral-hygiene-related mobile apps is presented in Supplementary Tables S3–S9. All of the selected apps were technically working. Each of the nine apps has a different developer. Even if one criteria of inclusion was free of charge, only two apps were fully free of charge, two required in-app purchases, and the others required a connected device to complete functioning. Depending on the stores, the number of downloads for each app was different. The most downloaded app on the two platforms was Oral-B (>1,000,000 downloads) and Disney Magic Timer by Oral-B (>5,000,000 downloads).

The characteristics of the nine oral-hygiene-related apps are described in Table 1. The apps focused on the increase of happiness or wellbeing (9/9, 100%), on behavior change and entertainment (8/9, 88.9%), and on goal-setting (7/9, 77.8%). They used information and education as theoretical background or strategies, as the levers, enabling users to achieve the objectives set by the app. Six apps were usable by persons older than 13 years old, whereas one app was for all ages and two apps were for children under 12 years old. Technically, 55.6% of apps sent reminders, and 33.3% allowed password protection and required login.

Seven of the nine apps focused on the quality of dental brushing (Colgate Connect, Disney Magic Timer by Oral-B, Kolibree, *Mimizaur se brosse les dents*, Oral-B, Preventeeth, and Truthbrush). Among them, five apps targeted persons higher than 13 years old (Colgate Connect, Kolibree, Oral-B, Preventeeth, and Truthbrush) and two targeted children under 12 years old (Disney Magic Timer by Oral-B and *Mimizaur se brosse les dents*). The *Santé Orale-SOHDEV* app introduced daily brushing and presented the course of a consultation with the dentist. This app targeted people with autism or other pervasive developmental disorders. The Dental Hygiene Mastery NBDHE app aimed to prepare for the National Board Dental Hygienist License and, to do so, provided oral hygiene questions and answers.

### 3.3. Reliability of the Evaluation

The reliability was good to excellent for engagement (section A) (ICC 0.88, 95% CI 0.82–0.92), for functionality (section B) (ICC 0.70, 95% CI 0.53–0.82), aesthetics (section C) (ICC 0.86, 95% CI 0.77–0.93), and information quality (section D) (ICC 0.94, 95% CI 0.91–0.96) sections individually. The reliability was excellent for the subjective quality (section E) (ICC 0.87, 95% CI 0.80–0.93) and for the section related to the specificities (section F) (ICC 0.84, 95% CI 0.76–0.90).

### 3.4. Qualitative Assessment of Oral-Health-Related Mobile App Content

**Figure 2:**
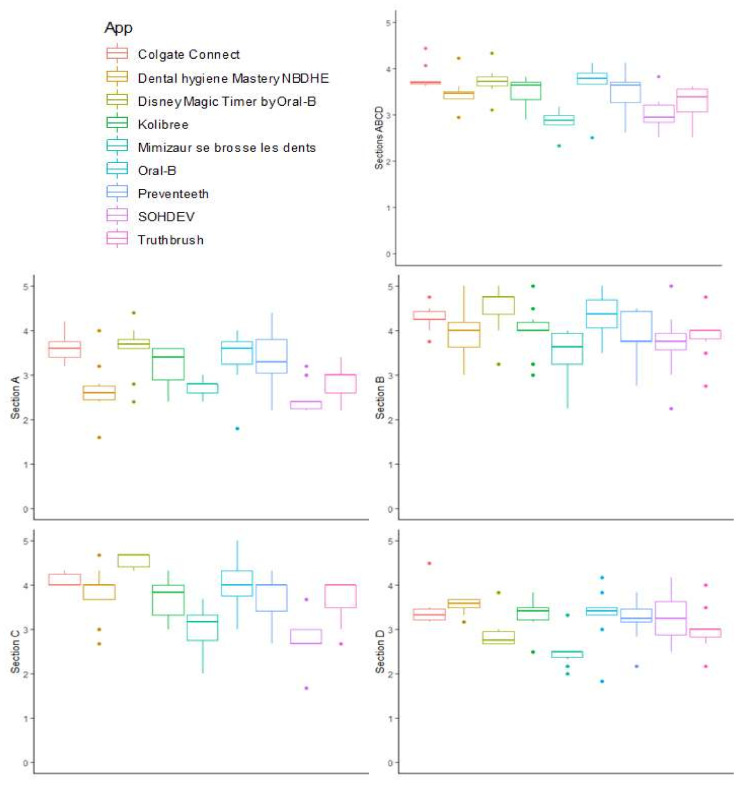
Qualitative evaluation of oral-health-related mobile apps. Section A: engagement; section B: functionality; section C: aesthetics; section D: information.

The best overall quality MARS scores (sections A, B, C, D) (Figure 2 and Supplementary Tables S10 and S11) were obtained by Oral-B (3.4 ± 0.97), Colgate Connect (3.20 ± 0.63), and Preventeeth (3.10 ± 1.1); whereas the worst overall quality MARS scores were obtained by *Mimizaur se brosse les dents* (1.80 ± 0.79) and Kolibree (2.30 ± 0.82).

The engagement (section A) scores ranged from a mean of 2.48 ± 0.34 for Santé Orale-SOHDEV to a mean of 3.62 ± 0.30 for Colgate Connect. The functionality (section B) scores ranged from a mean of 3.50 ± 0.54 for *Mimizaur se brosse les dents* to a mean of 4.5 ± 0.54 for Disney Magic Timer by Oral-B. The aesthetics (section C) scores ranged from a mean of 2.73 ± 0.50 for Santé Orale-SOHDEV to a mean of 4.56 ± 0.16 for Disney Magic Timer by Oral-B. The information quality (section D) scores ranged from a mean of 2.48 ± 0.34 for *Mimizaur se brosse les dents* to a mean of 3.53 ± 0.17 for Dental Hygiene Mastery NBDHE. Among the four different sections of the quality score, the functionality and the aesthetics scores were the highest for all the nine apps tested.

**Figure 3:**
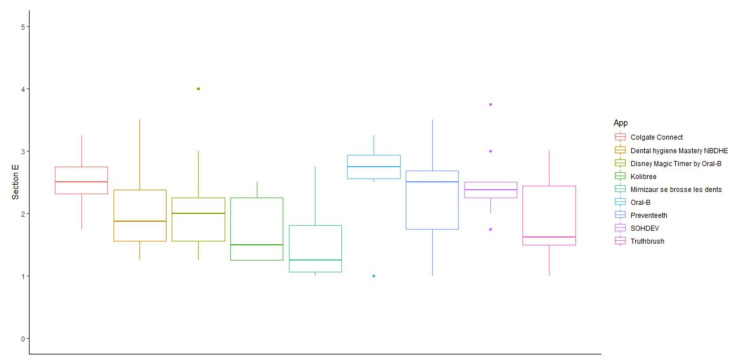
Subjective qualitative evaluation of oral-health-related mobile apps (section E).

The subjective quality scores (section E) (Figure 3) ranged from a mean of 1.5 ± 0.61 for *Mimizaur se brosse les dents* to a mean of 2.62 ± 0.61 for Oral-B.

### 3.5. Assessing the Content Specificity of Oral-Health-Related Mobile Applications

**Figure 4:**
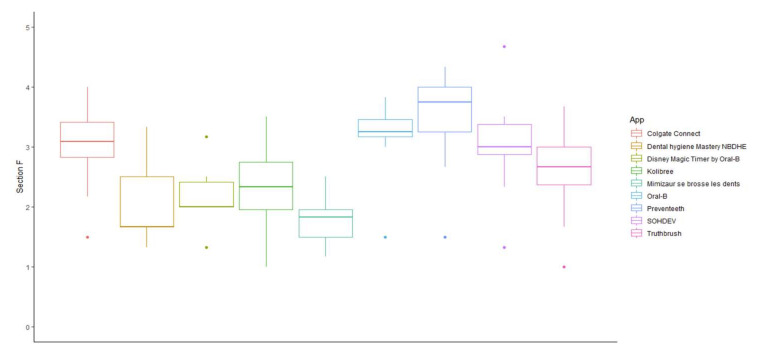
App-specific scores of oral-health-related mobile apps (section F).

The scores analyzing the specificity (section F) of the content of the apps (Figure 4) ranged from 3.46 ± 0.84 for Preventeeth, to 1.78 ± 0.47 for *Mimizaur se brosse les dents*. The best specificity quality scores were obtained by Preventeeth, Oral-B (3.15 ± 0.62), Santé Orale-SOHDEV (3.01 ± 0.85), Colgate Connect (2.94 ± 0.72), and Truthbrush (2.55 ± 0.75); whereas the worst specificity quality scores were obtained by *Mimizaur se brosse les dents*, Dental Hygiene Mastery NDBHE (2.08 ± 0.74), Disney Magic Timer by Oral-B (2.34 ± 0.56), and Kolibree (2.31 ± 0.69).

### 3.6. Strengths and Weaknesses of Each Oral-Health-Related Mobile Application

The app-specific score (section F) was always higher than the subjective quality score (section E), which was always lower than the overall quality MARS score (Supplementary Tables S10 and S11).

The heatmap (Table 2) analyzes the strengths and the weakness of each app. The strength of the quality score was functionality (section B) for all applications, except for Oral-B’s Disney Magic Timer, for which the strength of the quality score was aesthetics (section C). The weakness of the quality score was the information (section D) for five apps (Colgate Connect, Disney Magic Timer by Oral-B, *Mimizaur se brosse les dents,* Oral-B, and Preventeeth) and the engagement (section A) for four apps (Dental Hygiene Mastery NDBHE, Kolibree, *Santé Orale—SOHDEV,* and Truthbrush). For all apps except *Santé Orale—SOHDEV*, the worst scores in the quality score (sections A, B, C, and D) were observed for credibility (item 18). *Santé Orale—SOHDEV* obtained the worst score for interactivity.

For app subjective quality (section E), the best score was observed for the overall star rating (item 23) for all the apps except *Santé Orale—SOHDEV,* for which the best score was observed for the item indicating if the oral health professional would recommend the app (item 20). The worst score, for all nine apps, was observed for the item indicating whether people would be willing to pay for this app (item 22).

The specificity scores were very close to items for the same app, except for Dental Hygiene Mastery NDBHE and Oral-B.

### 3.7. Comparison of MARS Score and Star Rating

The correlation between the mean quality MARS score and the subjective item 23 (“What is your overall star rating of the app?”) was considered to be good (*r* = 0.62, *p <* 0.001). The overall quality MARS score was generally higher than the scores of the subjective item 23. This overall star rating MARS score (item 23) was also lower than the star rating score from the iOS or Android app stores except for Colgate Connect, for which the star rating in the Android was similar to the overall star rating MARS (Table 3).

## 4. Discussion

Screening of oral-health-related mobile apps available in the French Apple App and Google Play App stores yielded nine apps. In another study, screening of the United States Apple App and Google Play stores yielded 33 oral-health-related mobile apps (12 for Android, 11 for iOS, and 10 for both Android and iOS) [42]. Their criteria of inclusion were different because they included apps in English, and targeted adult consumers. They also limited their sample to the most popular and highly rated apps on each platform [42]. Sharif et al. (2019) [43] and Parker et al. (2019) [44] included 20 patient-focused oral hygiene apps available on the United Kingdom Apple App Store and Google Play store. Chen et al. (2021) screened apps focusing on dental caries self-management behaviors, including oral hygiene, dietary intake, and fluoride usage [45]. They performed their research in the Australian iTunes and Google Play App stores and included 18 apps in their study.

Although all the included apps focused on oral hygiene, two were specifically adapted for children. The Disney Magic Timer by Oral-B was associated with a better MARS score than *Mimizaur se brosse les dents*. This ranking does not preclude the implementation of good oral hygiene practices. For the adult users, the apps had a mean MARS score higher than 3.0 and did not reveal any preferred apps. Four of the nine apps required the purchase of a powered toothbrush to fully function. This may prevent app adherence and, thus, acquisition of proper oral hygiene even if some features or indications were usable, such as the tooth brushing time or the area to be cleaned. Due to the fact that no advantage of daily powered toothbrushing as compared to daily manual toothbrushing was seen with respect to oral hygiene or clinical parameters [46], the development of oral-health-related mobile apps focusing on quality tooth brushing without the need of powered toothbrush would be necessary. Both apps for children under 12 years old were timers and focused on the tooth brushing time, but not on the technical aspect of tooth brushing. The technical aspect should not be neglected because the study of Eidenhardt et al. (2021) concluded that regardless of age, adolescents between 10 and 15 years old divided their brushing time unevenly between internal, external, and occlusal surfaces. The internal surfaces, in particular, were largely neglected, as no age group spent more than 15.8% of total brushing time on them [47]. None of the nine apps included focused on diet, tobacco, and alcohol, which are significant risk factors for oral disease. In their study analyzing mobile apps for oral health promotion from the US iOS and Android stores, Tiffany et al. (2018) concluded that when topics such as diet, tobacco, and alcohol were addressed in the app, then the content was typically brief and sometimes included information that might be considered counterproductive to the goal of improving users’ health behavior [48].

Users and health care professionals have different expectations of applications, which results in different ratings [49]. First, the star ratings in the iOS and Android stores were higher than the MARS-F quality scores. Star rating and user comments are valuable to users because they provide insight into the effectiveness and popularity of apps [50], but star rating does not provide objective assessment of quality. In contrast to the study of Tiffany et al. (2018) [42], the star rating was not an inclusion criterion. This criterion would have been too restrictive and not objective because the apps included in our study were evaluated by no, or very few, people. For example, Truthbrush was evaluated by one rater in the iOS store and by no one in the Android store. Second, even when using scientific validated assessment scales, users and professionals have provided different scores. For example, the app Mimizavr Clean Teeth in the iOS and Android stores, which corresponds to *Mimizaur se brosse les dents* in the French stores, was evaluated with the scale MARS for users. Users rated the quality as 4.8 [51], whereas oral health professionals, in our study, rated it as 1.8. For the Disney Magic Timer by Oral-B, the users rated it 4.3 [51], whereas in our study, the oral health professionals rated it as 2.7. This discordance of views between professionals and users can have several reasons. The MARS and uMARS [52] questionnaires are similar but have slight differences. For example, the information section of uMARS has only four questions, while the MARS version has seven questions. The question on the credibility of the app is based on the users’ feelings for uMARS and on publications in the case of MARS. Moreover, professionals base their judgment on the content of the app while users are more interested in the design, attractiveness, or even by the gamification system related to the app.

Oral health professionals did not adhere to the use of applications related to oral hygiene. They rated the quality of the apps as good; they evaluated that many of the apps could have a positive impact on the user’s knowledge, attitudes, and intentions to change, as well as the probability of a real change in their oral hygiene behavior. However, they reported that they did not intend to use the app often in the next 12 months, even though it was relevant to them, and that they would not pay for the app. Similar conclusions were observed in a study analyzing nutrition-related mobile apps [53]. The strength of the apps, except for Disney, was the functionality, as observed in the study of Sharif et al. [43] that evaluated oral hygiene apps using MARS. However, Sharif et al. [43] concluded that the weakness of the apps was related to information, which was verified for only five apps in our study. The information quality section scored poorly due to the credibility of the apps. In fact, the source of the information was identified, but its validity or reliability was questionable (e.g., a commercial enterprise with a vested interest). In addition, the reviewers had difficulty assessing the level of scientific evidence and therefore selected “N/A The application has not been tested” in most cases. To the best of our knowledge, only five of the nine apps (Colgate Connect [42], Oral-B [42], Disney Magic Timer by Oral-B [45,51], Kolibree [42], and *Mimizaur se brosse les dents* [51]) are indexed in PubMed. To increase the credibility score and, thus, the information score, new scientific evidence is needed. Randomized controlled trials need to be undertaken to determine the clinical effectiveness of these applications as well as economic evaluations [54]. Demonstrated clinical effectiveness through improved measurable health outcomes will facilitate wider adoption of this and other effective applications in clinical practice. The integration of mobile apps and follow-up by health professionals in therapeutic programs could help to change behavior. However, the ethical issues surrounding the use of health data and the development of apps for commercial purposes should be considered.

This study has several limitations. First, only oral-hygiene-related mobile apps available on both Apple and Android French stores were included. Other stores, such as the Windows Phone Store, the Huawei store, the BlackBerry store, or the Samsung store, could have been investigated, which could have highlighted other applications. Second, the French version of the MARS was used because this scale is the most commonly used in the scientific literature for mobile health app evaluation to date [45,55,56,57,58,59]. However, other scales, such as ENLIGHT, could have been used [60]. Evaluation with other validated scales could lead to similar or divergent results. Third, the assessment was conducted by oral health professionals, whereas MHAs are intended for the general public. In future research, a comparison of our results with the user evaluation version of MARS would be interesting [52] to see the agreement between the opinion of professionals and users. Fourth, the applications included were very different in terms of objectives and target audience, making score comparisons difficult. Fifth, three of the nine reviewers were already familiar with the Oral-B application prior to the study, which may have influenced their opinions.

## 5. Conclusions

This observational study demonstrated that even if the oral health professionals positively evaluated the quality of the hygiene-related mobile apps available on both Apple and Android French stores, they are less assertive concerning their impact on the user’s knowledge, attitudes, and intentions to change, as well as the likelihood of actual change in the oral hygiene behavior. Further studies are needed to assess changes in oral hygiene behavior of app users. Thus, with evidence that mobile health apps can help prevent disease, it is important that developers focus on the information in these apps and that the effectiveness of these apps is scientifically proven, to change the opinion of professionals.

## Figures and Tables

**Table 1:** Characteristics of the nine oral-hygiene-related mobile apps.

Characteristic	App (n = 9), n (%) ^1^
**Focus or target**	
Increase happiness or wellbeing	9 (100)
Behavior change	8 (88.9)
Entertainment	8 (88.9)
Goal-setting	7 (77.8)
**Theoretical background or strategies**	
Assessment	6 (66.7)
Feedback	5 (55.6)
Information/Education	9 (100)
Monitoring/Tracking	6 (66.7)
Goal-setting	7 (77.8)
Advice/Tips/Strategies/Skills training	7 (77.8)
CBT–Behavioral (positive events)	6 (66.7)
Gratitude	7 (77.8)
Strengths-based	5 (55.6)
**Age group**	
Children (under 12 years)	3 (33.3)
Adolescents (13–17 years)	7 (77.8)
Young adults (18–25 years)	7 (77.8)
Adults	7 (77.8)
**Technical aspects of app**	
Allows sharing (Facebook, Twitter, etc.)	2 (22.2)
Allows password-protection	3 (33.3)
Requires login	3 (33.3)
Sends reminders	5 (55.6)

^1^ More than one could be applicable; therefore, percentages do not add to 100%.

**Table 2:** Heatmap of the average scores per item and per app. The colors are related to the scores and go from yellow (1: worst score) to green (5: best score).

	Colgate Connect	Dental Hygiene Mastery NBDHE	Disney magic Timer by Oral-B	Kolibree	*Mimizaur se brosse les dents*	Oral-B	Preventeeth	SOHDEV	Truthbrush
**Section A**									
Item 1—Entertainment	3.2	2.5	4.3	3.5	3.5	3.5	3.9	3.1	2.7
Item 2—Interest	3.3	3.1	4	3.5	3.3	3.9	4	3.3	3
Item 3—Customisation	3.8	2.9	2.9	3	1.9	2.9	3	1.4	2.9
Item 4—Interactivity	3.8	2.1	2.7	2.9	1.3	2.8	2.4	1	2.6
Item 5—Target group	4	2.8	4	3.3	3.6	3.8	3.7	3.6	3
**Section B**									
Item 6—Performance	4.6	4.2	4.6	4	4	4.4	3.5	3.9	4.1
Item 7—Ease of use	4.1	3.8	4.6	3.8	3.7	4.3	4.3	4	3.5
Item 8—Navigation	4.2	3.9	4.7	4.1	2.8	4.1	4	3.3	3.9
Item 9—Gestural design	4.3	4	4.2	4.1	3.5	4.7	3.9	3.6	4
**Section C**									
Item 10—Layout	4	3.9	4.1	4	3.3	4	3.7	2.7	3.8
Item 11—Graphics	4.4	3.9	4.9	3.7	2.9	4.3	3.8	2.7	3.7
Item 12—Visual appeal	3.9	3.6	4.7	3.5	2.8	3.9	3.5	2.8	3.6
**Section D**									
Item 13—Accuracy	3.9	3.7	3.2	3.7	3.6	3.9	3.4	3.4	3.2
Item 14—Goals	3.9	4	3.1	3.6	2.2	3.5	3.1	3	3.2
Item 15—Quality of information	4	3.6	3	3.7	2.3	3.6	3.8	3.4	3.4
Item 16—Quantity of information	3.6	4	2.3	3.8	2.3	3.7	3.2	3.4	3.7
Item 17—Visual information	4	4	4.5	4	2.8	3.9	4	3.5	3.4
Item 18—Credibility	1.2	1.9	1.2	1.2	1.7	1.4	1.7	2.9	1.1
**Section E**									
Item 20—Recommendations	3	2	2.2	1.5	1.5	2.9	2.8	3.1	1.6
Item 21—Usage	3.1	2.2	2.5	2.2	1.7	3.2	2.2	2.7	2.2
Item 22—Price	1	1.4	1.2	1	1	1	1.2	1.2	1
Item 23—Overall rating	3.2	2.7	2.7	2.3	1.8	3.4	3.1	2.9	2.8
**Section F**									
Awareness	2.9	1.8	2.3	2	1.9	3.7	3.7	2.8	2.8
Knowledge	2.9	4.1	1.7	2.2	1.1	2.9	3.3	3	2.4
Attitude	3.1	1.8	2.5	2.8	2.1	3.7	3.6	3	2.6
Intention to change	3.1	1.8	2.5	2.2	1.9	3.2	3.4	3.2	2.3
Help seeking	2.7	1.5	2	2	1.6	1.8	3.4	2.9	2.6
Behaviour change	3.2	1.5	2.4	2.7	2.1	3.6	3.4	3.2	2.6

**Table 3:** MARS overall star rating, overall quality MARS score, star rating in the iOS App Store, and star rating in the Android app store of the nine oral-hygiene-related mobile apps.

App Name	MARS Overall Star Rating (Item 23)	Overall Quality MARS Score	Star Rating in the iOS App Store (No. of Raters)	Star Rating in the Android App Store (No. of Raters)
Colgate Connect	3.2 ± 0.63	3.7 ± 0.26	4.3 (104)	3.1 (148)
*Dental Hygiene Mastery NBDHE*	2.7 ± 0.67	3.4 ± 0.35	NA	NA
Disney Magic Timer by Oral-B	2.7 ± 0.94	3.7 ± 0.30	4.3 (974)	4.4 (55,000)
Kolibree	2.3 ± 0.82	3.5 ± 0.29	4.1 (28)	3.5 (173)
*Mimizaur se brosse les dents*	1.8 ± 0.78	2.8 ± 0.22	4.5 (22)	4.6 (661)
Oral-B	3.4 ± 0.96	3.7 ± 0.45	4.6 (10,989)	4.5 (67,000)
Preventeeth	3.1 ± 1.10	3.4 ± 0.43	5 (18)	NA
Santé orale—SOHDEV	2.9 ± 0.73	3.0 ± 0.46	5 (1)	4.6 (9)
Truthbrush	2.8 ± 0.78	3.2 ± 0.36	5 (1)	NA

## Data Availability

The data presented in this study are available on request from the corresponding author.

## Supplementary Materials

The following supporting information can be downloaded at: https://www.mdpi.com/article/10.3390/ijerph19127293/s1, Table S1: STROBE Statement—checklist of items that should be included in reports of observational studies; Table S2: Characteristics of the raters, the hardware, and the software used; Table S3: Developer, scare rating, and paid content of the nine oral-health-related mobile apps; Table S4: Brief description of the nine oral-health-related mobile apps included in the study; Table S5: Targets of the nine oral-health-related mobile apps included in the study; Table S6: Theoretical background and strategies of the nine oral-health-related mobile apps included in the study; Table S7: Affiliations of the nine oral-health-related mobile apps included in the study; Table S8: Age group of the nine oral-health-related mobile apps included in the study; Table S9: Technical aspects of the nine oral-health-related mobile apps included in the study; Table S10: Mobile App Rating Scale (MARS) scoring by section; Table S11: Mobile App Rating Scale (MARS) scoring by items.
