# Supplementary material for: Oral-Hygiene-Related Mobile Apps in the French App Stores: Assessment of Functionality and Quality
Source: Int J Environ Res Public Health. 2022 Jun 14;19(12):7293. doi: 10.3390/ijerph19127293 (PMC9223428; doi:10.3390/ijerph19127293)
Supplement: Supplementary file 1 [file ijerph-19-07293-s001.zip › ijerph-1746154-supplementary.pdf]

Table S1. STROBE Statement—checklist of items that should be included in reports of observational studies.

	Item No	Recommendation	Page No
**Title and abstract**	1	(*a*) Indicate the study's design with a commonly used term in the title or the abstract	1
		(*b*) Provide in the abstract an informative and balanced summary of what was done and what was found	1
**Introduction**			
Background/rationale	2	Explain the scientific background and rationale for the investigation being reported	1-2
Objectives	3	State specific objectives, including any prespecified hypotheses	2
**Methods**			
Study design	4	Present key elements of study design early in the paper	2
Setting	5	Describe the setting, locations, and relevant dates, including periods of recruitment, exposure, follow-up, and data collection	2-3
Participants	6	(*a*) *Cohort study*—Give the eligibility criteria, and the sources and methods of selection of participants. Describe methods of follow-up<br>*Case-control study*—Give the eligibility criteria, and the sources and methods of case ascertainment and control selection. Give the rationale for the choice of cases and controls<br>*Cross-sectional study*—Give the eligibility criteria, and the sources and methods of selection of participants	3
		(*b*) *Cohort study*—For matched studies, give matching criteria and number of exposed and unexposed<br>*Case-control study*—For matched studies, give matching criteria and the number of controls per case	NA
Variables	7	Clearly define all outcomes, exposures, predictors, potential confounders, and effect modifiers. Give diagnostic criteria, if applicable	3
Data sources/ measurement	8*	For each variable of interest, give sources of data and details of methods of assessment (measurement). Describe comparability of assessment methods if there is more than one group	*3-4*
Bias	9	Describe any efforts to address potential sources of bias	*3-4*
Study size	10	Explain how the study size was arrived at	3
Quantitative variables	11	Explain how quantitative variables were handled in the analyses. If applicable, describe which groupings were chosen and why	3-4
Statistical methods	12	(*a*) Describe all statistical methods, including those used to control for confounding	3-4
		(*b*) Describe any methods used to examine subgroups and interactions	3-4
		(*c*) Explain how missing data were addressed	NA
		(*d*) *Cohort study*—If applicable, explain how loss to follow-up was addressed<br>*Case-control study*—If applicable, explain how matching of cases and controls was addressed<br>*Cross-sectional study*—If applicable, describe analytical methods taking account of sampling strategy	NA
		(*e*) Describe any sensitivity analyses	NA

:ontinued on next page

**Results**			
Participants	13*	(a) Report numbers of individuals at each stage of study—eg numbers potentially eligible, examined for eligibility, confirmed eligible, included in the study, completing follow-up, and analysed	4
		(b) Give reasons for non-participation at each stage	4
		(c) Consider use of a flow diagram	NA
Descriptive data	14*	(a) Give characteristics of study participants (eg demographic, clinical, social) and information on exposures and potential confounders	4
		(b) Indicate number of participants with missing data for each variable of interest	NA
		(c) *Cohort study*—Summarise follow-up time (eg, average and total amount)	NA
Outcome data	15*	*Cohort study*—Report numbers of outcome events or summary measures over time	*NA*
		Case-control study—Report numbers in each exposure category, or summary measures of exposure	*NA*
		Cross-sectional study—Report numbers of outcome events or summary measures	*NA*
Main results	16	(*a*) Give unadjusted estimates and, if applicable, confounder-adjusted estimates and their precision (eg, 95% confidence interval). Make clear which confounders were adjusted for and why they were included	4-10
		(*b*) Report category boundaries when continuous variables were categorized	NA
		(*c*) If relevant, consider translating estimates of relative risk into absolute risk for a meaningful time period	NA
Other analyses	17	Report other analyses done—eg analyses of subgroups and interactions, and sensitivity analyses	NA
**Discussion**			
Key results	18	Summarise key results with reference to study objectives	10-11
Limitations	19	Discuss limitations of the study, taking into account sources of potential bias or imprecision. Discuss both direction and magnitude of any potential bias	11-12
Interpretation	20	Give a cautious overall interpretation of results considering objectives, limitations, multiplicity of analyses, results from similar studies, and other relevant evidence	12-13
Generalisability	21	Discuss the generalisability (external validity) of the study results	12-13
**Other information**			
Funding	22	Give the source of funding and the role of the funders for the present study and, if applicable, for the original study on which the present article is based	NA

*Give information separately for cases and controls in case-control studies and, if applicable, for exposed and unexposed groups in cohort and cross-sectional studies.

Note: An Explanation and Elaboration article discusses each checklist item and gives methodological background and published examples of transparent reporting. The STROBE checklist is best used in conjunction with this article (freely available on the Web sites of PLoS Medicine at http://www.plosmedicine.org/, Annals of Internal Medicine at http://www.annals.org/, and Epidemiology at http://www.epidem.com/). Information on the STROBE Initiative is available at www.strobe-statement.org.

Table S2. Characteristics of the raters, the hardware and the software used.

	Rater 1	Rater 2	Rater 3	Rater 4	Rater 5	Rater 6	Rater 7	Rater 8	Rater 9	Rater 10
**Year of experience as dentist**	41	24	Student	Student	13	6	4	10	Student	6
**Affiliation**	Liberal and Hospital	Liberal and Hospital	University	University	Hospital	Liberal and Hospital	Liberal	Hospital	University	Liberal and Hospital
**Scope of activity**	Periodontist - Public health	General practice - Public health	NA[1]	NA[1]	Individual oral prevention	Public health	General practice	Public health and Oral surgery	NA[1]	Periodontist - Public health
**Hardware (phone)**	iphone 12 mini	iphone 8	iphone	iphone	iPhone 11	Samsung galaxy s21	iPhone 12	iPhone SE	Oppo Reno 4	Samsung galaxy A45
**Software (iOS or Android)**	iOS	iOS	iOS	iOS	iOS	Android	iOS	iOS	Android	Android

[1]NA: Not applicable

Table S3. Developer, Scare rating and Paid content of the 9 oral health-related mobile apps.

App name	Developer	Rating in the iOS app store (Nb of raters)	Rating in the Android app store (Nb of raters)	Paid Content
Colgate Connect	Colgate-Palmolive Compagny	4.3 (104)	3.1 (148)	Free App - Need connected device to fully function
Dental Hygiene Mastery NBDHE	Higher Learning Technologies	NA	NA	Free with in-app purchases
Disney magic Timer by Oral-B	Disney	4.3 (974)	4.4 (55000)	Free App
Kolibree	Kolibree	4.1 (28)	3.5 (173)	Free App - Need connected device to fully function
Mimizaur se brosse les dents	Shorokh Natalya	4.5 (22)	4.6 (661)	Free with in-app purchases
Oral-B	P&G Productions	4,6 (10989)	4.5 (67000)	Free App - Need connected device to fully function
Preventeeth	Alexandre Gambiez	5 (18)	NA	Free with in-app purchases
Santé orale - SOHDEV	Santé orale handicap dépendance vulnerab	5 (1)	4.6 (9)	Free App
Truthbrush	Candibell	5 (1)	NA	Free App - Need connected device to fully function

Table S4. Brief description of the 9 oral health-related mobile apps included in the study.

App name	Brief description of app
Colgate Connect	This application aims to improve the quality of dental brushing by increasing the brushing time and brushing areas. By connecting the electric toothbrush, a personalized follow-up is proposed.
Dental Hygiene Mastery NBDHE	This application offers questions and answers on oral hygiene based on dental hygienists' exam questions.
Disney magic Timer by Oral-B	This application aims to increase tooth brushing time in children by using Disney characters and fun games.
Kolibree	This application aims to improve the quality of dental brushing by increasing the brushing time and brushing areas. By connecting the electric toothbrush, a personalized follow-up is proposed.
Mimizaur se brosse les dents	This application aims to learn how to brush your teeth through different cartoons whose main character is Mimizaur.
Oral-B	This application aims to improve the quality of dental brushing by increasing the brushing time and brushing areas. By connecting the electric toothbrush, a personalized follow-up is proposed.
Preventeeth	This application aims to teach how to take care of your teeth, how to brush better, and how to avoid dental problems. It allows personalized and individual learning regardless of the toothbrush used. It proposes exercises and calculates a score related to the risk of having dental problems.
Santé Orale - SOHDEV	This application has been designed to reduce the number of visits to the dentist and to introduce daily brushing for people with autism or other pervasive developmental disorders (PDD).
Truthbrush	This application aims to track daily events related to the toothbrush and represents them as a graph over time. The application displays the time of brushing, the duration and the coverage. Truthbrush knows the difference between brushing teeth, applying toothpaste and cleaning the brush. The app makes it easy to set goals and rewards, set up alerts and even share brushing data with your dentist. It works fully when attached to the Truthbrush device, which fits any manual or powered toothbrush.

Table S5. Targets of the 9 oral health-related mobile apps included in the study.

	**Colgate Connect**	**Dental Hygiene Mastery**	**Disney magic Timer by Oral-B**	**Kolibree**	***Mimizaur se brosse les dents***	**Oral-B**	**Preventeeth**	***Santé orale - SOHDEV***	**Truthbrush**
**Focus: what the app targets (select all that apply)**									
Increase Happiness/Well-being	X	X	X	X	X	X	X	X	X
Mindfulness/Meditation/Relaxation									
Anxiety/Stress									
Behaviour Change	X		X	X	X	X	X	X	X
Goal Setting	X	X	X	X		X	X		X
Entertainment	X		X	X	X	X	X	X	X
Relationships									
Physical health									

Table S6. Theoretical background and strategies of the 9 oral health-related mobile apps included in the study.

	**Colgate Connect**	**Dental Hygiene Mastery NBDHE**	**Disney magic Timer by Oral-B**	**Kolibree**	***Mimizaur se brosse les dents***	**Oral-B**	**Preventeeth**	***Santé orale - SOHDEV***	**Truthbrush**
**Theoretical background/Strategies (all that apply)**									
Assessment	X	X		X		X	X		X
Feedback	X	X		X		X			X
Information/Education	X	X	X	X	X	X	X	X	X
Monitoring/Tracking	X	X	X	X		X			X
Goal setting	X	X	X	X		X	X		X
Advice /Tips /Strategies /Skills training	X	X		X		X	X	X	X
CBT - Behavioral (positive events)	X		X	X		X	X		X
CBT – Cognitive (thought challenging)									
ACT - Acceptance commitment therapy									
Mindfulness/Meditation									
Relaxation									
Gratitude	X	X	X	X		X	X		X
Strengths based	X			X		X	X		X

Table S7. Affiliations of the 9 oral health-related mobile apps included in the study.

	**Colgate Connect**	**Dental Hygiene Mastery NBDHE**	**Disney magic Timer by Oral-B**	**Kolibree**	***Mimizaur se brosse les dents***	**Oral-B**	**Preventeeth**	***Santé orale - SOHDEV***	**Truthbrush**
Unknown									
Commercial	X	X	X	X	X	X	X		X
Government									
Non-governmental association								X	

Table S8. Age group of the 9 oral health-related mobile apps included in the study.

	**Colgate Connect**	**Dental Hygiene Mastery NBDHE**	**Disney magic Timer by Oral-B**	**Kolibree**	***Mimizaur se brosse les dents***	**Oral-B**	**Preventeeth**	***Santé orale - SOHDEV***	**Truthbrush**
**Age group (all that apply)**									
Children (under 12)			X		X			X	
Adolescents (13-17)	X	X		X		X	X	X	X
Young Adults (18-25)	X	X		X		X	X	X	X
Adults	X	X		X		X	X	X	X

Table S9. Technical aspects of the 9 oral health-related mobile apps included in the study.

	**Colgate Connect**	**Dental Hygiene Mastery NBDHE**	**Disney magic Timer by Oral-B**	**Kolibree**	***Mimizaur se brosse les dents***	**Oral-B**	**Preventeeth**	***Santé orale - SOHDEV***	**Truthbrush**
**Technical aspects of app (all that apply)**									
Allows sharing (Facebook, Twitter, etc.)				X					X
Has an app community									
Allows password-protection	X	X							X
Requires login	X	X							X
Sends reminders	X	X		X		X			X
Needs web access to function									X

Table S10. Mobile App Rating Scale (MARS) scoring by section.

App Name	Data	Section A	Section B	Section C	Section D	Section ABCD	Section E	Section F
Colgate Connect	Mean	3.62	4.3	4.09	3.43	3.78	2.57	2.98
	SD	0.30	0.30	0.15	0.39	0.26	0.45	0.72
Dental Hygiene Mastery NBDHE	Mean	2.68	3.97	3.80	3.53	3.43	2.07	2.08
	SD	0.61	0.55	0.59	0.17	0.35	0.78	0.74
Disney magic Timer by Oral-B	Mean	3.58	4.52	4.56	2.88	3.72	2.15	2.23
	SD	0.57	0.54	0.16	0.35	0.30	0.81	0.56
Kolibree	Mean	3.24	4.00	3.72	3.33	3.52	1.75	2.31
	SD	0.44	0.56	0.46	0.35	0.29	0.55	0.69
Mimizaur se brosse les dents	Mean	2.72	3.50	2.99	2.48	2.86	1.5	1.78
	SD	0.21	0.54	0.52	0.34	0.22	0.61	0.47
Oral-B	Mean	3.38	4.37	4.06	3.33	3.70	2.62	3.15
	SD	0.62	0.45	0.56	0.61	0.45	0.61	0.62
Preventeeth	Mean	3.4	3.92	3.66	3.20	3.49	2.32	3.46
	SD	0.66	0.54	0.58	0.44	0.43	0.76	0.84
Santé orale - SOHDEV	Mean	2.48	3.70	2.73	3.26	3.05	2.47	3.01
	SD	0.34	0.72	0.68	0.53	0.46	0.55	0.83
Truthbrush	Mean	2.84	3.87	3.70	3.00	3.26	1.90	2.55
	SD	0.37	0.50	0.50	0.48	0.36	0.67	0.75

Table S11. Mobile App Rating Scale (MARS) scoring by items.

App Name	Data	Section A					Section B				Section C			Section D						
		Item 1	Item 2	Item 3	Item 4	Item 5	Item 6	Item 7	Item 8	Item 9	Item 10	Item 11	Item 12	Item 13	Item 14	Item 15	Item 16	Item 17	Item 18	Item 19
Colgate Connect	Mean	3.20	3.3	3.8	3.8	4	4.6	4.1	4.2	4.3	4	4.4	3.9	3.9	3.9	4	3.6	4	1.2	X
	SD	0.42	0.48	0.63	0.42	0.47	0.51	0.56	0.42	0.48	0	0.51	0.31	0.73	0.56	0	0.69	0.47	0.63	X
Dental Hygiene Mastery NBDHE	Mean	2.5	3.1	2.9	2.1	2.8	4.2	3.8	3.9	4	3.9	3.9	3.6	3.7	4	3.6	4	4	1.9	X
	SD	1.26	1.10	0.87	0.73	1.13	0.63	0.91	0.73	0.47	0.87	0.56	0.69	0.67	0.47	0.51	0	0.47	0.56	X
Disney magic Timer by Oral-B	Mean	4.3	4.00	2.9	2.7	4	4.6	4.6	4.7	4.2	4.1	4.9	4.7	3.2	3.1	3	2.3	4.5	1.2	X
	SD	0.82	0.81	0.87	1.05	0.47	0.96	0.51	0.48	0.63	0.31	0.31	0.48	1.03	0.31	0.47	0.82	0.70	0.63	X
Kolibree	Mean	3.5	3.5	3	2.9	3.3	4	3.8	4.1	4.1	4	3.7	3.5	3.7	3.6	3.7	3.8	4	1.2	X
	SD	0.70	0.97	1.24	0.87	0.94	0.47	0.91	0.73	0.56	0.66	0.48	0.70	0.67	0.51	0.67	0.78	0.47	0.63	X
Mimizaur se brosse les dents	Mean	3.5	3.3	1.9	1.3	3.6	4	3.7	2.8	3.5	3.3	2.9	2.8	3.6	2.2	2.3	2.3	2.8	1.7	X
	SD	0.70	0.48	0.56	0.67	0.69	0.47	1.05	1.13	0.52	0.82	0.87	0.42	0.69	0.42	0.67	0.67	0.78	0.48	X
Oral-B	Mean	3.5	3.9	2.9	2.8	3.8	4.4	4.3	4.1	4.7	4	4.3	3.9	3.9	3.5	3.6	3.7	3.9	1.4	X
	SD	0.97	0.99	0.87	0.78	0.78	0.69	0.67	0.56	0.48	0.66	0.67	0.73	1.10	0.70	0.69	0.48	1.10	0.84	X
Preventeeth	Mean	3.9	4	3	2.4	3.7	3.5	4.3	4	3.9	3.7	3.8	3.5	3.4	3.1	3.8	3.2	4	1.7	X
	SD	0.99	0.94	1.24	0.84	0.67	0.97	0.67	0.47	0.31	0.67	0.78	0.70	0.96	0.31	0.78	0.78	0.47	0.67	X
Santé orale - SOHDEV	Mean	3.10	3.3	1.4	1	3.6	3.9	4	3.3	3.6	2.7	2.7	2.8	3.4	3	3.4	3.4	3.5	2.9	X
	SD	0.87	0.67	0.51	0	0.69	1.19	0.47	1.05	0.84	1.33	0.82	0.42	0.96	0.47	0.69	0.69	0.97	0.73	X
Truthbrush	Mean	2.7	3	2.9	2.6	3	4.1	3.5	3.9	4	3.8	3.7	3.6	3.2	3.2	3.4	3.7	3.4	1.1	X
	SD	0.82	0.81	0.87	0.51	0.47	0.31	1.17	0.56	0.47	0.42	0.67	0.69	0.91	0.42	0.96	0.82	0.84	0.31	X

App Name	Data	Section E				Mobile app specificities					
		Item 20	Item 21	Item 22	Item 23	Awareness	Knowledge	Attitudes	Intention to change	Help seeking	Behavior change
Colgate Connect	Mean	3	3.1	1	3.2	2.9	2.9	3.1	3.1	2.7	3.2
	SD	0.47	1.19	0	0.63	0.87	1.19	0.87	0.56	1.05	0.78
Dental Hygiene Mastery NBDHE	Mean	2	2.2	1.4	2.7	1.8	4.1	1.8	1.8	1.5	1.5
	SD	0.66	1.61	0.84	0.67	1.22	0.73	1.13	0.63	0.97	0.70
Disney magic Timer by Oral-B	Mean	2.2	2.5	1.2	2.7	2.3	1.7	2.5	2.5	2	2.4
	SD	0.78	1.50	0.63	0.94	1.05	0.67	0.70	0.97	0.81	0.69
Kolibree	Mean	1.5	2.2	1	2.3	2	2.2	2.8	2.2	2	2.7
	SD	0.70	1.54	0	0.82	0.81	0.63	0.91	0.91	0.81	0.82
Mimizaur se brosse les dents	Mean	1.5	1.7	1	1.8	1.9	1.1	2.1	1.9	1.6	2.1
	SD	0.84	1.25	0	0.78	0.56	0.31	0.99	0.97	0.51	0.87
Oral-B	Mean	2.9	3.2	1	3.4	3.7	2.9	3.7	3.2	1.8	3.6
	SD	0.73	0.91	0	0.96	0.82	0.73	0.67	0.91	0.78	0.96
Preventeeth	Mean	2.8	2.2	1.2	3.1	3.7	3.3	3.6	3.4	3.4	3.4
	SD	0.91	1.13	0.63	1.10	0.82	1.33	0.69	0.96	1.07	1.07
Santé orale - SOHDEV	Mean	3.1	2.7	1	2.9	2.8	3	3	3.2	2.9	3.2
	SD	0.56	0.82	0	0.73	1.03	0.47	0.66	1.13	1.19	1.13
Truthbrush	Mean	1.6	2.2	1	2.8	2.8	2.4	2.6	2.3	2.6	2.6
	SD	0.96	1.47	0	0.78	0.78	0.84	1.07	0.94	0.96	0.84
